# The equity gap in precision dosing: a cross-sectional analysis of hospital-wide antimicrobial therapeutic drug monitoring implementation

**DOI:** 10.3389/fpubh.2026.1748173

**Published:** 2026-03-31

**Authors:** Bo Yang, Bei Zheng, Juan Wang, Hong Jiang, Wenjuan Yang, Hongxian Xu, Bin Han, Chuanwei Xin, Meiling Zhang, Yuexing Tu

**Affiliations:** 1 Department of Pharmacy, Tongde Hospital of Zhejiang Province, Hangzhou, Zhejiang, China; 2 Department of Clinical Laboratory Science, Tongde Hospital of Zhejiang Province, Hangzhou, Zhejiang, China; 3 Department of Pharmacy, Hangzhou Linping Hospital of Traditional Chinese Medicine, Hangzhou, Zhejiang, China; 4 Department of Critical Care Medicine, Tongde Hospital of Zhejiang Province, Hangzhou, Zhejiang, China

**Keywords:** antimicrobial agents, antimicrobial stewardship, implementation science, public health, Therapeutic Drug Monitoring (TDM)

## Abstract

**Background:**

Therapeutic Drug Monitoring (TDM) is pivotal for optimizing antimicrobial efficacy and managing drug-resistant infections. However, its real-world implementation patterns, particularly the heterogeneity in initial hospital-wide application within large Chinese general hospitals, have not been systematically evaluated.

**Methods:**

This retrospective study was conducted in a representative Chinese tertiary hospital to assess its hospital-wide antimicrobial TDM program launched in June 2024. The analysis included 2,316 plasma drug concentration measurements from 1,928 inpatients between October and December 2024, primarily evaluated based on trough concentrations.

**Results:**

The study revealed significant inter-departmental heterogeneity in TDM implementation. Utilization was high in intensive care units (e.g., EICU: 91.95%) and infection-related departments, but critically low in non-intensive care settings (e.g., Pediatrics: 0%). Overall, only 50.3% of drug concentrations were within the therapeutic range, with time-dependent antimicrobials (e.g., piperacillin attainment: 31.47%) performing significantly worse than concentration-dependent agents (e.g., moxifloxacin attainment: 86.09%). Subtherapeutic concentrations were predominantly clustered in hematology patients, while supratherapeutic concentrations were concentrated in ICUs. The study also demonstrated the inadequacy of a trough concentration-based monitoring strategy for addressing the complex pharmacokinetics in critically ill patients.

**Conclusion:**

This study provides a critical snapshot of the heterogeneous implementation and systemic inequities in the initial rollout of a hospital-wide antimicrobial TDM program. The findings highlight a significant gap between specialized technique and equitable public health practice. Addressing these implementation disparities requires tailored strategies, multidisciplinary collaboration, and refined monitoring approaches to optimize antimicrobial stewardship and ensure all patients benefit from precision dosing.

## 1 Introduction

Antimicrobial resistance has emerged as one of the most pressing public health threats of the 21st century. According to the World Health Organization (WHO), one in six bacterial infections worldwide is now resistant to antibiotic treatments, with resistance rates rising in over 40% of monitored pathogen-antibiotic combinations between 2018 and 2023 (1). Global estimates indicate that bacterial AMR was directly responsible for approximately 1.14 million deaths in 2021 alone, contributing to nearly five million deaths annually when considering associated mortality (2). If current trends persist, drug-resistant infections could claim up to 10 million lives per year by 2050, with an estimated cumulative economic cost of 100 trillion USD (3). Low- and middle-income countries, including regions within China, bear a disproportionate share of this burden (4).

Therapeutic Drug Monitoring (TDM) is a critical component of antimicrobial stewardship (AMS), playing a pivotal role in optimizing antibiotic efficacy, particularly against complex infections and drug-resistant pathogens (5, 6). Its effective implementation is a public health priority in the global effort to combat antimicrobial resistance (AMR).

Despite this recognized priority, the translation of TDM from theoretical ideal into consistent real-world practice remains a significant challenge, particularly within large and diverse healthcare systems such as China’s (7). Understanding this implementation gap is critical for designing effective stewardship programs. This study was conducted in a representative Chinese tertiary care hospital that launched a pioneering, hospital-wide TDM program in June 2024. This initiative offers a unique opportunity to dissect the early-phase implementation of a critical public health tool. This study is a purely observational assessment of routine care practices and does not involve any intervention beyond standard clinical protocols.

By mapping the real-world implementation landscape of a multi-drug TDM program across an entire hospital during its critical inception period, this purely observational study aims to systematically document practice patterns and identify systemic gaps, rather than to primarily assess pharmacokinetic efficacy. This “implementation gap” analysis serves as a necessary first step and a detailed situational assessment; its findings can inform the design of targeted, equitable interventions and policies to improve access to precision dosing. Ultimately, understanding and addressing these early-stage challenges is crucial for translating TDM from a niche tool into a robust component of hospital-wide antimicrobial stewardship and public health defense against resistance.

## 2 Methods

### 2.1 Study design and patients

We conducted a retrospective, single-center, observational study to evaluate the early-phase rollout of a hospital-wide antimicrobial TDM program. This study was purely descriptive, analyzing existing TDM practices without implementing any changes to routine clinical care. This analysis included all hospitalized patients who underwent antimicrobial TDM at our institution between October and December 2024. The post-implementation period (the fourth quarter of 2024) was selected for this initial assessment to allow for workflow stabilization, providing a more reliable snapshot of the program’s early performance. Patients were excluded if their TDM records were incomplete (e.g., missing demographic information or unverifiable sampling time) or if the plasma concentration measurement failed quality control standards. The study protocol was approved by the Ethics Review Committee of Tongde Hospital of Zhejiang Provincial (approval no. 2025160-JY). Informed consent was waived due to the retrospective nature of the study.

### 2.2 TDM program implementation and workflow

The hospital-wide TDM program, launched in June 2024 as a multidisciplinary antimicrobial stewardship initiative, was guided by an institutional policy that encouraged the principle of “test when indicated” for a broad range of antimicrobials. This practice was particularly promoted for drugs with a narrow therapeutic index, potential toxicity, or significant pharmacokinetic variability, encompassing glycopeptides (e.g., vancomycin, norvancomycin), azole antifungals (e.g., voriconazole, fluconazole), oxazolidinones (e.g., linezolid), tigecycline, fluoroquinolones (e.g., moxifloxacin, levofloxacin), and key beta-lactams (e.g., piperacillin-tazobactam, ceftazidime-avibactam, cefoperazone-sulbactam, meropenem, imipenem). The operational workflow involved attending physicians making testing decisions, nursing staff responsible for blood sampling, and the clinical laboratory which performed analysis on weekdays (providing next-day results) while accepting samples on weekends for preprocessing. Clinical pharmacists took the primary role in interpreting TDM results and recommending dose adjustments in consultation with physicians. Upon implementation, comprehensive training on the rationale and procedures for TDM was delivered to relevant clinicians and pharmacists.

### 2.3 Data collection and analytical methods

In this study, we collected all available plasma concentration measurements of antimicrobial agents along with patient demographic data. The plasma concentrations were determined using high-performance liquid chromatography (Jasper MPLC system) coupled with tandem mass spectrometry (SCIEX Triple Quad 4500MD). In line with initial program feasibility and existing clinical routines, trough concentration (C_min_) was employed as the primary evaluation metric. The therapeutic plasma concentration ranges for antimicrobial agents were established based on previously reported literature data and adjusted according to the specific circumstances of our research center (Table 1). The lower limit of detection (LOD) for each assay is specified below.

**Table 1:** Therapeutic ranges (C_min_) and detection limits for antimicrobial agents.

Antimicrobial agent (source formulation)	Therapeutic range (C_min_ μg/mL)	LOD (μg/mL)	References
Piperacillin (piperacillin-tazobactam)	22.5–150	1.0	(34)
Ceftazidime (ceftazidime-avibactam)	16–64	1.0	(24)
Cefoperazone (cefoperazone-sulbactam)	20.5–89.12	5.0	(35)
Meropenem	2–45	1.0	(36)
Imipenem	2–20	0.4	(37)
Vancomycin	10–20	0.8	(38)
Norvancomycin	10–20	0.8	(38)
Linezolid	2–7	0.6	(39)
Tigecycline	0.13–0.87	0.02	(40)
Moxifloxacin	0.6–3.2	0.16	(41)
Levofloxacin	1–4	0.4	(41)
Fluconazole	10–20	0.8	(42)
Voriconazole	0.5–5	0.2	(43)
Caspofungin	>1	0.05	(44)

## 3 Results

### 3.1 Basic characteristics of therapeutic drug monitoring

Between October 2024 and December 2024, a total of 2,316 plasma antimicrobial concentration samples were collected from 1,928 hospitalized patients. The most frequently monitored agents were cefoperazone (527 samples, 22.75%), piperacillin (518 samples, 22.37%) and moxifloxacin (338 samples, 14.59%). Voriconazole and ceftazidime exhibited the highest frequency of repeated monitoring, with 33.33% (19/57) and 30.56% (11/36) of patients undergoing ≥2 measurements, respectively. However, the vast majority of patients (71.93%) received only one TDM measurement during the study period, indicating that sustained, dynamic monitoring was not yet a standard practice. Detailed data are summarized in Table 2.

**Table 2:** Demographic and TDM monitoring characteristics of patients.

Antimicrobial agent (source formulation) piperacillin (piperacillin-tazobactam)	Sex (M/F)	Age (years, mean ± SD)	Total samples (*n*)	Monitoring frequency [*n* (%)]			
				1 time	2 times	3 times	>3 times
Ceftazidime (ceftazidime-avibactam)	235/178	66.54 ± 18.33	518	342 (82.81)	54 (13.08)	7 (1.69)	10 (2.42)
Cefoperazone (cefoperazone-sulbactam)	25/11	70.22 ± 17.31	60	25 (69.44)	4 (11.11)	4 (11.11)	3 (8.33)
Antimicrobial agent (source formulation)	257/147	65.11 ± 17.48	527	331 (81.93)	49 (12.13)	14 (3.47)	10 (2.47)
Meropenem	129/106	67.68 ± 15.60	285	195 (82.98)	32 (13.62)	6 (2.55)	2 (0.85)
Imipenem	3/2	74.00 ± 16.48	5	5 (100.00)	-	-	-
Vancomycin	27/18	60.31 ± 19.18	53	38 (84.44)	6 (13.33)	1 (2.22)	
Norvancomycin	3/4	65.29 ± 15.74	7	7 (100.00)	-	-	-
Linezolid	45/29	66.54 ± 16.31	95	55 (74.32)	18 (24.32)	-	1 (1.35)
Tigecycline	15/9	72.83 ± 11.78	27	22 (91.67)	1 (4.17)	1 (4.17)	-
Moxifloxacin	154/181	54.19 ± 17.90	338	332 (99.10)	3 (0.90)	-	-
Levofloxacin	98/104	53.64 ± 20.34	208	197 (97.52)	4 (1.98)	1 (0.50)	-
Fluconazole	20/19	70.26 ± 15.61	51	31 (79.49)	4 (10.26)	4 (10.26)	-
Voriconazole	30/27	61.20 ± 15.56	86	38 (66.67)	11 (19.30)	6 (10.53)	2 (3.51)
Caspofungin	25/27	63.54 ± 14.50	56	48 (92.31)	4 (7.69)	-	-

### 3.2 Distribution of plasma drug concentrations

Among 2,316 antimicrobial plasma concentration measurements, 1,165 (50.30%) were within the therapeutic range. Subtherapeutic levels (detectable but below the therapeutic range) were observed in 445 samples (19.21%), while 346 samples (14.94%) were below the lower limit of detection (LOD). Supratherapeutic concentrations accounted for 360 samples (15.54%), indicating a substantial risk of concentration-dependent toxicity.

Notably, the target attainment rates revealed stark, class-specific disparities with direct implications for antimicrobial stewardship. Concentration-dependent agents such as caspofungin (98.21%), moxifloxacin (86.09%), and voriconazole (80.23%) demonstrated high target attainment. In stark contrast, time-dependent antimicrobials, the backbone of many empiric regimens, performed poorly: piperacillin (31.47%), ceftazidime (23.33%), vancomycin (37.74%), norvancomycin (14.29%), and linezolid (16.84%) all exhibited alarmingly low rates of target attainment. This systemic failure to optimize time-dependent antibiotics poses a dual threat: compromising individual patient outcomes and fostering the selection of resistant pathogens at a public health level. Detailed results are presented in Table 3.

**Table 3:** Therapeutic drug monitoring outcomes for 14 antimicrobial agents.

Antimicrobial agent (source formulation)	Mean concentration (μg/mL, mean ± SD)	Below LOD [*n* (%)]	Subtherapeutic [*n* (%)]	Within range [*n* (%)]	Supratherapeutic [*n* (%)]
Piperacillin (piperacillin-tazobactam)	63.28 ± 78.50	168 (32.43)	133 (25.68)	163 (31.47)	54 (10.42)
Ceftazidime (ceftazidime-avibactam)	130.41 ± 99.73	-	5 (8.33)	14 (23.33)	41 (68.33)
Cefoperazone (cefoperazone-sulbactam)	57.56 ± 48.68	47 (8.92)	122 (23.15)	251 (47.63)	107 (20.30)
Meropenem	10.88 ± 12.15	93 (32.63)	30 (10.53)	155 (54.39)	7 (2.46)
Imipenem	26.38 ± 32.82	-	1 (20.00)	2 (40.00)	2 (40.00)
Vancomycin	16.50 ± 11.09	2 (3.77)	15 (28.30)	20 (37.74)	16 (30.19)
Norvancomycin	11.69 ± 9.11	-	5 (71.43)	1 (14.29)	1 (14.29)
Linezolid	12.08 ± 8.94	10 (10.53)	9 (9.47)	16 (16.84)	60 (63.16)
Tigecycline	0.59 ± 0.48	1 (3.70)	3 (11.11)	17 (62.96)	6 (22.22)
Moxifloxacin	1.24 ± 0.82	7 (2.07)	28 (8.28)	291 (86.09)	12 (3.55)
Levofloxacin	2.60 ± 3.64	15 (7.21)	76 (36.54)	87 (41.83)	30 (14.42)
Fluconazole	18.09 ± 12.64	-	11 (21.57)	24 (47.06)	16 (31.37)
Voriconazole	2.99 ± 2.13	3 (3.49)	6 (6.98)	69 (80.23)	8 (9.30)
Caspofungin	3.57 ± 2.25	-	1 (1.79)	55 (98.21)	-

### 3.3 Distribution of plasma drug concentrations relative to therapeutic ranges

**Figure 1:**
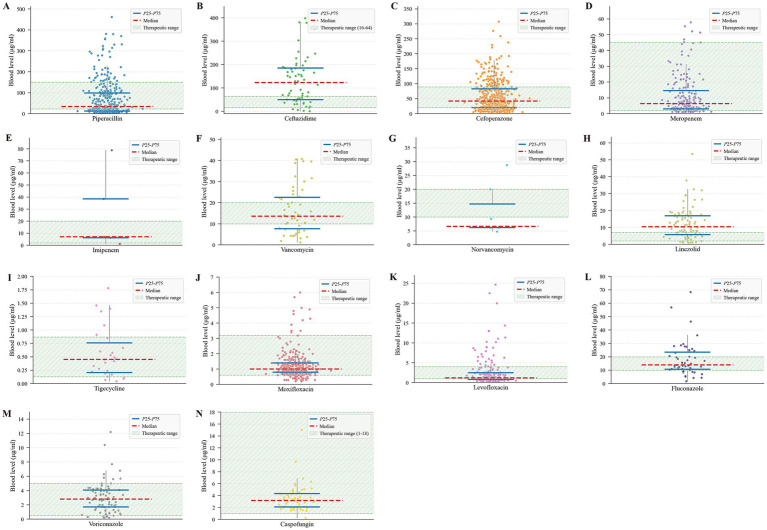
Distribution of plasma antimicrobial concentrations relative to recommended therapeutic windows. **(A)** Piperacillin. **(B)** Ceftazidime. **(C)** Cefoperazone. **(D)** Meropenem. **(E)** Imipenem. **(F)** Vancomycin. **(G)** Norvancomycin. **(H)** Linezolid. **(I)** Tigecycline. **(J)** Moxifloxacin. **(K)** Levofloxacin. **(L)** Fluconazole. **(M)** Voriconazole. **(N)** Caspofungin.

The 25th-75th percentiles (P25-P75) of plasma concentrations for ceftazidime, imipenem, and linezolid demonstrated significant upward shifts compared to their recommended therapeutic windows. Conversely, piperacillin, norvancomycin, and levofloxacin exhibited downward shifts in their P25-P75 ranges relative to the reference targets. Other monitored agents, including meropenem, moxifloxacin, and voriconazole, showed good alignment with literature-based therapeutic ranges. A detailed comparison of interquartile ranges (IQRs) and target windows is illustrated in Figure 1.

### 3.4 Departmental variations in TDM utilization

**Figure 2:**
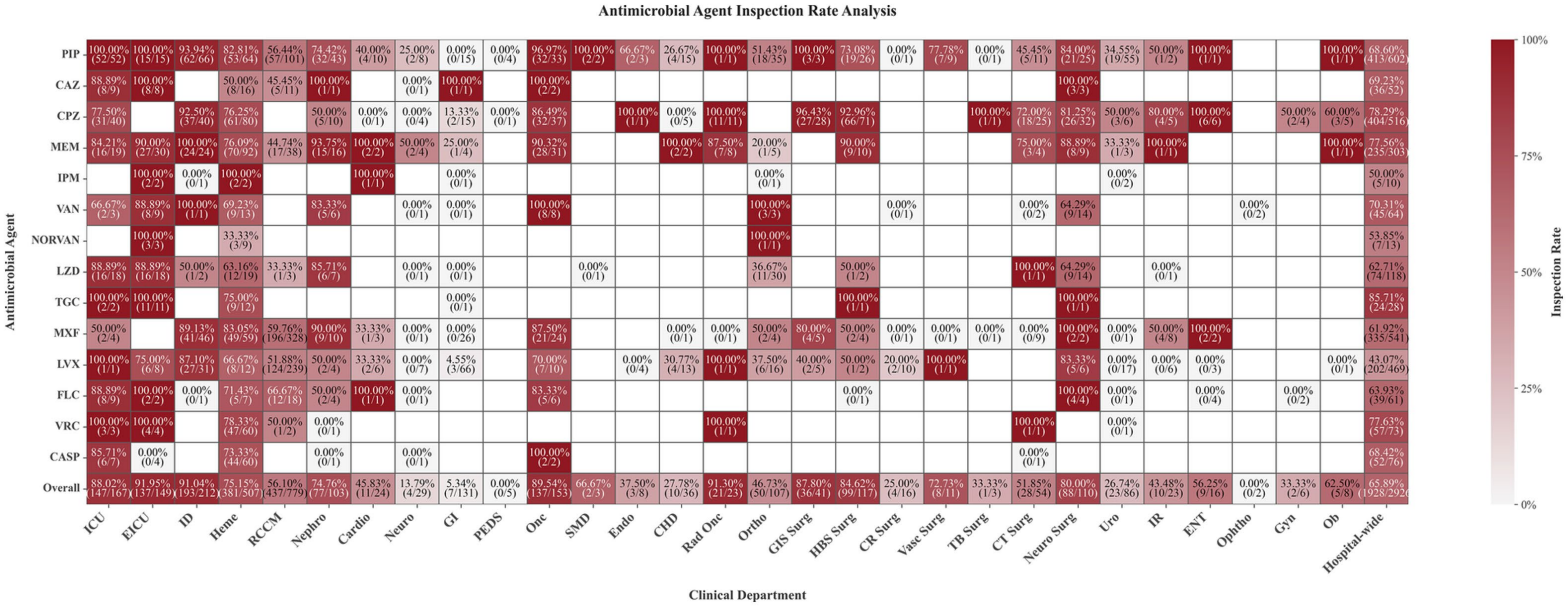
Departmental variations in TDM utilization rates for antimicrobial agents. PIP, Piperacillin; CAZ, Ceftazidime; CPZ, Cefoperazone; MEM, Meropenem; IPM, Imipenem; VAN, Vancomycin; NORVAN, Norvancomycin; LZD, Linezolid; TGC, Tigecycline (TGC); MXF, Moxifloxacin; LVF, Levofloxacin; FLC, Fluconazole; VORI, Voriconazole; CSP, Caspofungin; ICU, Intensive Care Unit; EICU, Emergency Intensive Care Unit; ID, Infectious Diseases Department; Heme, Hematology Department; RCCM, Respiratory and Critical Care Medicine; Nephro, Nephrology Department; Cardio, Cardiovascular Medicine Department; Neuro, Neurology Department; GI, Gastroenterology Department; PEDS, Pediatrics Department; Onc, Oncology Department; SMD, Sleep Medicine Department; Endo, Endocrinology Department; CHD, Cadre Health Care Department; Rad Onc, Radiation Oncology Department; Ortho, Orthopedics Department; GIS Surg, Gastrointestinal Surgery; HBS Surg, Hepatobiliary Surgery; CR Surg, Colorectal Surgery; Vasc Surg, Vascular Surgery; TB Surg, Thyroid and Breast Surgery; CT Surg, Cardiothoracic Surgery; Neuro Surg, Neurosurgery Department; Uro, Urology Department; IR, Interventional Radiology Department; ENT, Otolaryngology Department; Ophtho, Ophthalmology Department; Gyn, Gynecology Department; Ob, Obstetrics Department.

A profound disparity in TDM utilization was observed across clinical departments (Figure 2), reflecting systemic inequities in access to precision dosing. As anticipated, critical care settings such as Emergency Intensive Care Unit (EICU) (91.95%) and Intensive Care Unit (ICU) (88.02%) demonstrated near-universal integration of TDM into practice. For core antimicrobials, TDM utilization in these units frequently reached 100%, underscoring its role as a standard tool for balancing efficacy and toxicity in severely ill patients. Infection-focused departments, such as Infectious Diseases (91.04%), also exhibited high utilization, aligning with their mandate to manage complex and often drug-resistant infections.

Notably, high utilization rates were also observed in the Oncology (89.54%) and Radiation Oncology (91.30%) departments. This pattern likely reflects the heightened vulnerability of their patient populations, who are often immunocompromised due to underlying malignancy or cytotoxic therapies and are at high risk for severe infections. In this context, TDM is employed as a critical preventive strategy to ensure adequate antimicrobial exposure and manage complex drug–drug interactions, rather than stemming from a primary focus on infection management.

In stark contrast, non-intensive care departments showed critically low engagement. Most notably, the Pediatrics Department reported a 0% TDM utilization rate for multiple agents, indicating a complete absence of structured therapeutic monitoring for this vulnerable population. Similarly, the Gastroenterology Department recorded 0% utilization for key agents, and the Neurology Department’s overall utilization rate was only 13.79%. These findings suggest that current TDM practices may be failing entire patient groups in non-critical settings.

Surgical departments displayed moderate but variable TDM use. For instance, Vascular Surgery achieved a 77.78% utilization rate for piperacillin-tazobactam, whereas Thyroid/Endocrine Surgery recorded 0%. Such variability at the sub-specialty level underscores the need for tailored, discipline-specific TDM protocols.

Further analysis from the perspective of antimicrobial agents themselves revealed consistent patterns. Drugs with narrow therapeutic indices and well-established toxicity profiles, such as tigecycline (85.71%), voriconazole (77.63%), and vancomycin (70.31%), were among the top five with the highest TDM utilization rates. This reflects a targeted clinical strategy to mitigate specific risks, such as hepatotoxicity and nephrotoxicity, by optimizing exposure. Conversely, several frequently used agents like levofloxacin (43.07%) exhibited relatively low TDM uptake. This was particularly evident in non-intensive care settings such as Pediatrics and Gastroenterology, where monitoring for these agents was almost absent, potentially increasing the risks of ototoxicity or neuromuscular blockade.

These multi-level disparities, observed across both departments and drug classes, reflect the combined influence of clinical characteristics, drug-specific properties, and physician awareness on TDM implementation. To improve overall management, tailored monitoring strategies must be developed for different departments and antimicrobial agents.

### 3.5 Distribution of subtherapeutic drug concentrations across departments

**Figure 3:**
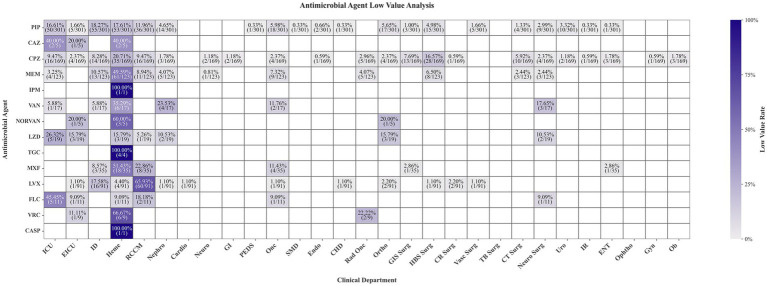
Distribution of subtherapeutic antimicrobial plasma concentrations across clinical departments.

Analysis of subtherapeutic antimicrobial concentrations (Figure 3) revealed significant interdepartmental heterogeneity in their distribution. Subtherapeutic samples were predominantly clustered in critical care and infection-related departments, likely attributable to their higher TDM utilization rates. Surprisingly, the hematology department emerged as a major contributor, accounting for the highest proportion of subtherapeutic samples across most monitored antimicrobial agents. This finding highlights a specific public health vulnerability: patients with hematologic malignancies, who are often immunocompromised and subject to complex pharmacokinetic alterations (e.g., hypoalbuminemia, drug interactions), are systematically experiencing suboptimal antibiotic exposure. This not only jeopardizes their survival but also creates an ideal environment for the emergence of resistant infections within this cohort.

### 3.6 Distribution of supratherapeutic drug concentrations across departments

**Figure 4:**
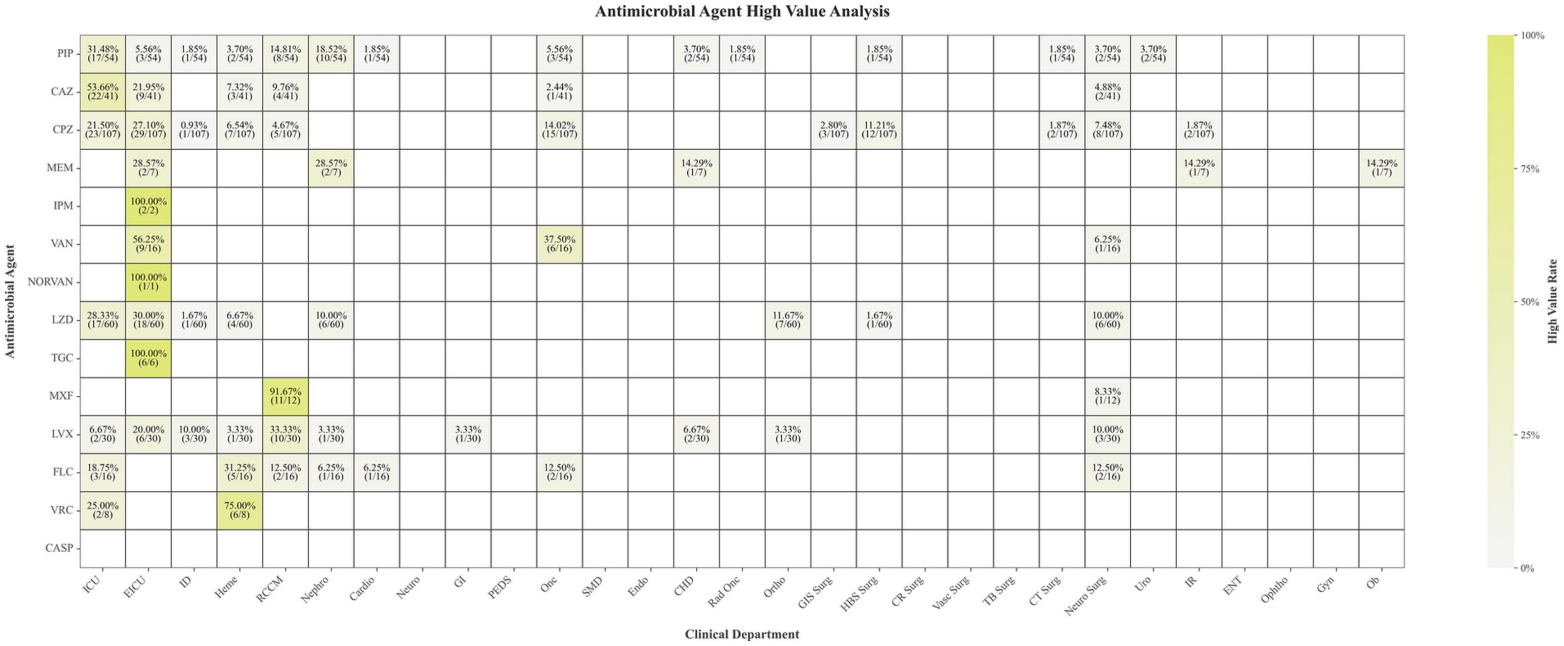
Distribution of supratherapeutic plasma drug concentrations across clinical departments.

Supratherapeutic antimicrobial concentrations were clustered predominantly in clinical areas managing the most vulnerable and complex patients (Figure 4). In the ICU, which accounted for 53.66% (22/41) of supratherapeutic ceftazidime samples, this pattern likely reflects a combination of impaired renal clearance in critically ill patients and empirical high-dose regimens targeting multidrug-resistant pathogens. Notably, tigecycline in the EICU exhibited a 22.22% (6/27) supratherapeutic rate despite 100% TDM compliance, underscoring the hepatotoxicity risks associated with its narrow therapeutic window. The Respiratory and Critical Care Medicine department contributed a high proportion of samples exceeding the reference therapeutic concentrations for quinolone agents, with 91.67% (11/12) for moxifloxacin and 33.33% (10/30) for levofloxacin. The Hematology department showed a significant contribution of supratherapeutic levels for antifungal agents, with 31.25% (5/16) for fluconazole and 75.00% (6/8) for voriconazole. Notably, the Oncology department accounted for 37.50% of vancomycin samples exceeding the reference therapeutic range, which may further compromise renal function in patients already at risk due to various chemotherapeutic agents.

## 4 Discussion

This study provides the first comprehensive landscape of a hospital-wide antimicrobial Therapeutic Drug Monitoring (TDM) program during its initial implementation phase in a large Chinese tertiary care hospital. As a purely observational study, it describes real-world practice patterns without intervention. The ancient adage by Paracelsus that “the right dose differentiates a poison from a remedy” finds direct validation in our data, which collectively demonstrate that current TDM practices often fail to ensure the “right dose” at a systemic level (8). Our analysis underscores three interrelated public health concerns: prevalent underexposure to critical antimicrobials, particularly time-dependent antibiotics; significant inter-departmental inequity in TDM utilization; and the inherent limitations of a trough concentration (C_min_)-based monitoring strategy. Collectively, these findings underscore that translating TDM from a specialized technique into an effective, equitable public health tool requires overcoming deeply rooted systemic challenges.

### 4.1 From technical adoption to system integration: gaps and inequities in implementation

The most striking finding is the profound and systemic inequity in TDM application across the hospital, from 91.95% in the EICU to 0% in Pediatrics. This represents a failure in the equitable distribution of a precision public health intervention within a single institution. High utilization in critical care, infectious diseases, and oncology reflects integration into workflows for managing complex patients and drug interactions (9, 10). Conversely, the near-absence in Pediatrics, Gastroenterology, and Neurology signals a major implementation gap. The complete lack of TDM in pediatrics is particularly alarming, stemming not from a lack of need but from systemic barriers: age-dependent pharmacokinetic variability, practical difficulties with blood sampling, and a lack of pediatric-specific protocols (11, 12).

This inequity extends to surgical disciplines, where utilization varied markedly, influenced by departmental culture and risk perception (13, 14). To bridge these gaps, a multifaceted strategy is required: pragmatic workflow simplification (e.g., microsampling in pediatrics), development of tailored clinical decision support tools, and enhanced interdisciplinary education (15, 16). A pragmatic approach involves multidisciplinary stewardship teams to guide dosing in non-critical care and surgical fields, ensuring precision medicine benefits all patients (17, 18).

### 4.2 Suboptimal target attainment: an underestimated public health risk

The suboptimal overall target attainment rate of 50.3% observed in our cohort represents a significant threat to public health. This concern is starkly illustrated by the pronounced disparity between antimicrobial classes. Time-dependent antibiotics, which are the cornerstone of empirical therapy for severe infections, exhibited alarmingly low success rates: piperacillin-tazobactam (31.47%), cefoperazone-sulbactam (47.63%), and ceftazidime-avibactam (23.33%). These figures are consistent with multicenter studies reporting *β*-lactam target attainment rates of 35.0–48.9% (19, 20). In sharp contrast, concentration-dependent agents such as moxifloxacin achieved an 86.09% attainment rate. This systemic under-exposure of first-line time-dependent agents presents a dual public health threat.

First, it directly fuels the antimicrobial resistance crisis. Persistent subtherapeutic exposure creates sustained selective pressure, fostering the emergence and spread of resistant pathogens (21). Second, our findings point to the inadequacy of the predominant trough concentration (C_min_)-based monitoring strategy as a key driver of this failure (22). For time-dependent agents like piperacillin, which require sustained concentration above a threshold, a single trough measurement is vulnerable to clinical workflow irregularities (e.g., delayed administration or sampling), potentially misrepresenting the true pharmacodynamic profile and leading to unnecessary dose escalations (23). Conversely, for drugs like ceftazidime, we observed a high rate of supratherapeutic concentrations (68.33%), indicating that empirical high-dose regimens, monitored solely by trough levels, may inadvertently increase toxicity risks (24, 25).

Therefore, moving beyond trough-based monitoring is a public health imperative. Adopting more descriptive pharmacokinetic metrics, such as area under the curve (AUC) estimation through limited sampling strategies (26) or real-time modeling software (27), is crucial to accurately optimize dosing, ensure therapeutic efficacy, and curb the advancement of antimicrobial resistance.

### 4.3 Pharmacokinetic alterations in high-risk populations: a call for targeted intervention

Our findings reveal that subtherapeutic and supratherapeutic concentrations are not randomly distributed but are clustered in specific, high-risk patient populations. This clustering underscores that antimicrobial exposure is profoundly influenced by the underlying pathophysiology of the patient, necessitating a move beyond one-size-fits-all dosing regimens.

The hematology department emerged as a significant hotspot for subtherapeutic concentrations (Figure 3). This can be attributed to a constellation of factors unique to this population: chronic immunosuppression, prevalent hypoalbuminemia, and a high burden of drug–drug interactions. For instance, hypoalbuminemia can increase the volume of distribution for hydrophilic antibiotics like *β*-lactams, causing total plasma concentrations to fall below the therapeutic range while the pharmacologically active free fraction may be even lower (28, 29). Studies have shown that hematology patients with hypoalbuminemia face a substantially higher risk of subtherapeutic piperacillin exposure (30). Without TDM-guided dose adjustments, this underexposure not only jeopardizes outcomes in a vulnerable cohort but also creates a reservoir for selecting resistant pathogens, amplifying a public health threat (21).

Conversely, the clustering of supratherapeutic concentrations in ICUs (Figure 4) highlights the tension between aggressive empiric therapy and altered pharmacokinetics in critical illness. Critically ill patients frequently exhibit impaired renal clearance or require extracorporeal support (e.g., CRRT), elevating risks of drug accumulation (31). The 22.22% supratherapeutic rate for tigecycline in the EICU, despite full TDM compliance, starkly illustrates the ongoing toxicity risk posed by narrow therapeutic windows (0.13–0.87 μg/mL) in unstable patients (32, 33).

These findings compel a paradigm shift from reactive monitoring to proactive, pre-emptive dose optimization for defined high-risk groups. For hematology patients, initial dosing regimens should account for predicted pharmacokinetic alterations and mandate early TDM. In the ICU, dynamic monitoring coupled with real-time assessment of organ function is essential. Integrating TDM into a broader framework of precision medicine that considers individual patient characteristics is the key to mitigating toxicity and curbing resistance in these sentinel populations.

### 4.4 Limitations and future perspectives

This study has several limitations. First, the pharmacokinetic assessment relied solely on trough concentrations (C_min_), without integrating key pharmacodynamic targets such as AUC/MIC or %T > MIC, which may limit accurate exposure characterization, especially for time-dependent agents. Second, the lack of patient outcome data precludes direct assessment of TDM’s impact on efficacy or safety. Third, as a single-center, short-term observational study, the findings may not reflect long-term effectiveness, seasonal variations, or generalizable implementation patterns across hospitals. Future studies should extend the observation period, integrate TDM data with patient-centered outcomes, and involve multicenter collaborations to validate the identified gaps and develop standardized protocols. Advancing toward AUC-guided precision dosing will be fundamental in solidifying TDM’s role as an essential public health strategy against antimicrobial resistance.

## 5 Conclusion

Our findings reveal that implementing an effective antimicrobial therapeutic drug monitoring program requires more than just technical capability. It demands a fundamental rethinking of how we integrate precision medicine into routine hospital care. The significant disparities we identified across departments, combined with the poor performance of essential time-dependent antibiotics, highlight systemic challenges that cannot be solved by simply adding another test to the clinical workflow. Instead, we must transform TDM into an intelligent partner in patient care. This means developing standardized yet flexible protocols that ensure equal access for all patients, creating smart tools that help clinicians make better dosing decisions, and building collaborative teams that bring specialized expertise directly to the bedside. By making TDM an integral part of a learning healthcare system, we can protect both current patients and our future antibiotic resources, turning this powerful tool into a genuine defense for public health.

## Data Availability

The raw data supporting the conclusions of this article will be made available by the authors, without undue reservation.
